# A novel approach to cytoarchitectonics: developing an objective framework for the morphological analysis of the cerebral cortex

**DOI:** 10.3389/fnana.2024.1441645

**Published:** 2024-08-12

**Authors:** Matija Vid Prkačin, Zdravko Petanjek, Ivan Banovac

**Affiliations:** ^1^Department of Anatomy and Clinical Anatomy, University of Zagreb School of Medicine, Zagreb, Croatia; ^2^Croatian Institute for Brain Research, University of Zagreb School of Medicine, Zagreb, Croatia

**Keywords:** prefrontal cortex, human, prediction model, neural network, cell classification

## Abstract

**Introduction:**

The cytoarchitectonic boundaries between cortical regions and layers are usually defined by the presence or absence of certain cell types. However, these cell types are often not clearly defined and determining the exact boundaries of regions and layers can be challenging. Therefore, in our research, we attempted to define cortical regions and layers based on clear quantitative criteria.

**Methods:**

We performed immunofluorescent anti-NeuN labelling on five adult human brains in three cortical regions—Brodmann areas (BA) 9, 14r, and 24. We reconstructed the cell bodies of 90,723 NeuN-positive cells and analyzed their morphometric characteristics by cortical region and layer. We used a supervised neural network prediction algorithm to classify the reconstructions into morphological cell types. We used the results of the prediction algorithm to determine the proportions of different cell types in BA9, BA14r and BA24.

**Results:**

Our analysis revealed that the cytoarchitectonic descriptions of BA9, BA14r and BA24 were reflected in the morphometric measures and cell classifications obtained by the prediction algorithm. BA9 was characterized by the abundance of large pyramidal cells in layer III, BA14r was characterized by relatively smaller and more elongated cells compared to BA9, and BA24 was characterized by the presence of extremely elongated cells in layer V as well as relatively higher proportions of irregularly shaped cells.

**Discussion:**

The results of the prediction model agreed well with the qualitative expected cytoarchitectonic descriptions. This suggests that supervised machine learning could aid in defining the morphological characteristics of the cerebral cortex.

## 1 Introduction

Meaningful research on cortical cytoarchitectonics started in 19th century with the introduction of Nissl staining, which enabled researchers to extensively study the microstructural organization of the cerebral cortex. Despite the widespread use of the Nissl method by many researchers (Meynert, 1867, 1868; Hammarberg, 1895; Flechsig, 1897), initial works on cytoarchitectonics lacked a truly systematic approach and there was no agreed upon histological division of the cerebral cortex. It was not until Brodmann (1909) developed his cytoarchitectonic map of the cerebral cortex, that research on the morphology and function of different cortical regions began to be more comparable and reproducible. After Brodmann, von Economo and Koskinas made the next great contribution to research on cytoarchitectonics by producing one of the most comprehensive analyses of the human cerebral cortex (von Economo and Koskinas, 1925; von Economo, 1927).

Following these fundamental works, further research was mostly focused on refining Brodmann’s map based on new insights. Some of the research was comparative in nature, with the aim of finding both histological and functional homology of cortical regions between different species, usually primates (Ongür and Price, 2000; Ongür et al., 2003). Most of the research was predominantly qualitative (Petrides and Pandya, 1999; Petrides et al., 2012), though some studies attempted to quantify certain types of specialized cells in specific cortical regions (Rivara et al., 2003; Fajardo et al., 2008; González-Acosta et al., 2018). Those studies were somewhat limited because the detection of such specialized cells, and therefore their quantification, was still based on arbitrarily decided qualitative criteria. There were also some attempts to define cortical regions based on quantitative criteria, such as cell density and laminar thickness (Rajkowska and Goldman-Rakic, 1995a,b). However, this research still used exclusively manual quantification. Recently, there have been attempts to use machine learning in delineating cortical layers, with promising results (Štajduhar et al., 2023).

Nowadays, precise localization is extremely important in neuroscience research, and determining the exact cortical regions and layers, in which the morphological and molecular characteristics will be analyzed, is the golden standard in the field. Interestingly, even today, cortical regions and layers are still predominantly delineated by individual experts based on their prior experience and referring to relevant literature. A potential limitation of such an approach could be a less reliable comparability of research conducted by different research groups.

Cortical regions and layers are usually defined by the presence or absence of certain cell types (Nieuwenhuys, 1994). At the same time, the morphometric and molecular characteristics of those cells are often not well defined or agreed upon, though there were some attempts at achieving more objective interneuron classifications (Mihaljević et al., 2014, 2018). Detailed characterization of different cell types is essential due to several reasons. Firstly, many comparative studies showed interspecies differences in morphological and molecular characteristics of certain cortical cell types, especially the different interneuron subpopulations (Džaja et al., 2014; Hladnik et al., 2014; Banovac et al., 2022). Secondly, clinically related studies showed that alterations in cortical neurons found in different neuropsychiatric disorders affect very specific neuron populations, typically in specific cortical regions and layers (Marín, 2012; Prkačin et al., 2023).

The aforementioned shows that spatial localization and precise morphological and molecular definition of different neuron types are essential for understanding the functional organization of the cerebral cortex and its potential alterations in neuropathology. However, since such research heavily relies on the qualitative analysis of experts in the field, it may be prone to interrater bias and thus lack adequate reproducibility. In addition, the training of experts and their analyses are typically time consuming. This means that the applicability of such methodology in clinical practice remains low, which reduces its translational value.

Therefore, in our research, we attempted to define different cell types, as well as cortical regions and layers based on clear quantitative criteria. We also assessed the morphological composition of different cortical regions and layers as well as compared laminar differences between regions. We focused on Brodmann areas (BA) 9, 14r and 24 due to the following: (1) these regions are part of the human prefrontal cortex (PFC), and as such play a key role in integration of information from other cortical regions, as well as being involved in higher cognitive functions in humans (Fuster, 2015); (2) although these three regions are part of the PFC, they are phylogenetically and functionally very different, which should be reflected in their cytoarchitectonics. The aim of our study is to provide a more objective way of defining morphological cell types and delineating specific cortical layers and regions.

## 2 Materials and methods

### 2.1 Study design

In order to achieve our aim of developing a more objective way of defining morphological cell types and delineating cortical regions and layers, we structured our research as follows.

We chose PFC tissue blocks containing BA9, BA14r and BA24 from 5 human specimens—for the general localization of these cortical regions we used the cytoarchitectonic maps by Brodmann (1909), von Economo and Koskinas (1925), and Ongür and Price (2000). We confirmed the locations of the cortical regions using Nissl staining (see Supplementary data). We then performed immunofluorescent anti-NeuN labelling on three histological sections in each cortical region (45 sections in total).

After imaging the histological slides, we determined the exact boundaries of the cytoarchitectonic regions and delineated the cortical layers by cross-referencing descriptions and histological depictions in relevant literature (Brodmann, 1909; von Economo and Koskinas, 1925; von Economo, 1927; Braak, 1980; Vogt et al., 1995; Rajkowska and Goldman-Rakic, 1995a; Petrides and Pandya, 1999; Ongür and Price, 2000; Ongür et al., 2003; von Economo and Triarhou, 2009; Petrides et al., 2012) (for a detailed description see Supplementary data).

We then reconstructed the cell bodies of NeuN-positive (NeuN^+^) cells in all 45 imaged slides and analyzed their morphometric parameters by cortical region and layer. We then additionally classified a representative sample of reconstructions into different morphological cell types using the work of von Economo and Koskinas as our main reference.

Finally, we used a neural network prediction algorithm to classify the rest of the reconstructions into morphological types, based on the input from the manual classification. We used this data to determine the provisionary proportions of various morphological cell types in different cortical regions and layers.

### 2.2 Brain tissue samples

We analyzed the brain samples of five male human subjects aged from 37 to 51 years with a postmortem delay of 6 to 11 h (Supplementary Table S1). The subjects had no medical history of neurological or psychiatric disorders and no signs of preagonal state at autopsy. The brain tissue is stored in the brain bank as a part of the Zagreb Neuroembryological Collection (Kostovic et al., 1991; Judaš et al., 2011).

The University of Zagreb School of Medicine Ethics Committee approved the tissue collection and research conduction (Approval Nos. 380-59-10106-14-55/152 and 380-59-10106-19-111/210). The information on the subject’s identity and history is anonymized, and the brain samples are coded, indicating only the cortical region and the subject’s age.

The brain tissue blocks were cut according to Talairach’s and Szikla (1980) coordinates. Tissue blocks included parts of the PFC encompassing the superior frontal gyrus (containing BA9), straight gyrus (containing BA14r), and anterior cingulate cortex (containing BA24) (Brodmann, 1909; von Economo and Koskinas, 1925; Ongür and Price, 2000; Banovac et al., 2020). The tissue was fixed by immersion in 4% paraformaldehyde for 24 h, then dehydrated in an ethanol cascade (70, 96, 100%), vitrificated in toluene, and finally embedded in paraffin (Sadeghipour and Babaheidarian, 2019). The tissue was then cut on a microtome into 20 μm-thick coronal slices (Sy and Ang, 2019) and mounted on VitroGnost Plus Ultra adhesive microscope slides (BioGnost, Zagreb, Croatia). The tissue was always cut perpendicular to the dome of the gyrus to avoid distortion of cell body morphology.

### 2.3 Immunofluorescence labeling

Immunofluorescence was performed according to previously established protocol for paraffin-embedded tissue (Zaqout et al., 2020; Banovac et al., 2022). Histological sections were first photobleached for 48 h using a LED light source (Neumann and Gabel, 2002; Sun et al., 2017) in order to reduce autofluorescence. The sections were deparaffinized and heat antigen retrieval was performed in citrate-based (pH 6.0) unmasking solution (Boenisch, 2005). Protein blocking was done by incubating the sections for 1 h at room temperature (RT) in normal donkey serum (NDS; Chemicon, United States) diluted 1:30 in permeabilization solution (0.3% Triton X-100 in 1X PBS; Sigma-Aldrich, United States). Afterwards, the sections were incubated overnight in an anti-NeuN primary antibody (rabbit polyclonal, Abcam, CN: ab104225, lot: GR3410699-1, RRID: AB_10711153, working dilution: 1:1,000) at 4°C and in an anti-rabbit secondary antibody (donkey conjugated anti-rabbit Alexa 546, Thermo Fisher (Invitrogen), CN: A10040, lot: 2128963, RRID: AB_2534016, working dilution: 1:1,000) for 2 h at RT. The sections were treated with TrueBlack^®^ Lipofuscin Autofluorescence Quencher (Biotium, United States) to further reduce autofluorescence (Banovac et al., 2019) and coverslipped with VectaMount Aqueous Mounting Medium (Vector Laboratories, United States).

Histological sections were imaged by a laser confocal microscope (Olympus FLUOVIEW FV3000RS, Japan) on high-power magnification and using Z-stack, visualizing the entire section thickness.

### 2.4 Morphometric analysis

The confocal images used in morphometric analysis were maximum Z projections, meaning that the entire section thickness was projected onto a single image.

Neuron cell bodies were included in the morphometric analysis only if they met all of the following criteria: (1) the cell was NeuN^+^, (2) the cell body was fully visible (i.e., it was not covered by another cell and was not visibly cut on the edge of the section), (3) the outline of the cell body was clearly distinguishable from the background (Supplementary Figure S1).

**Figure 1:**
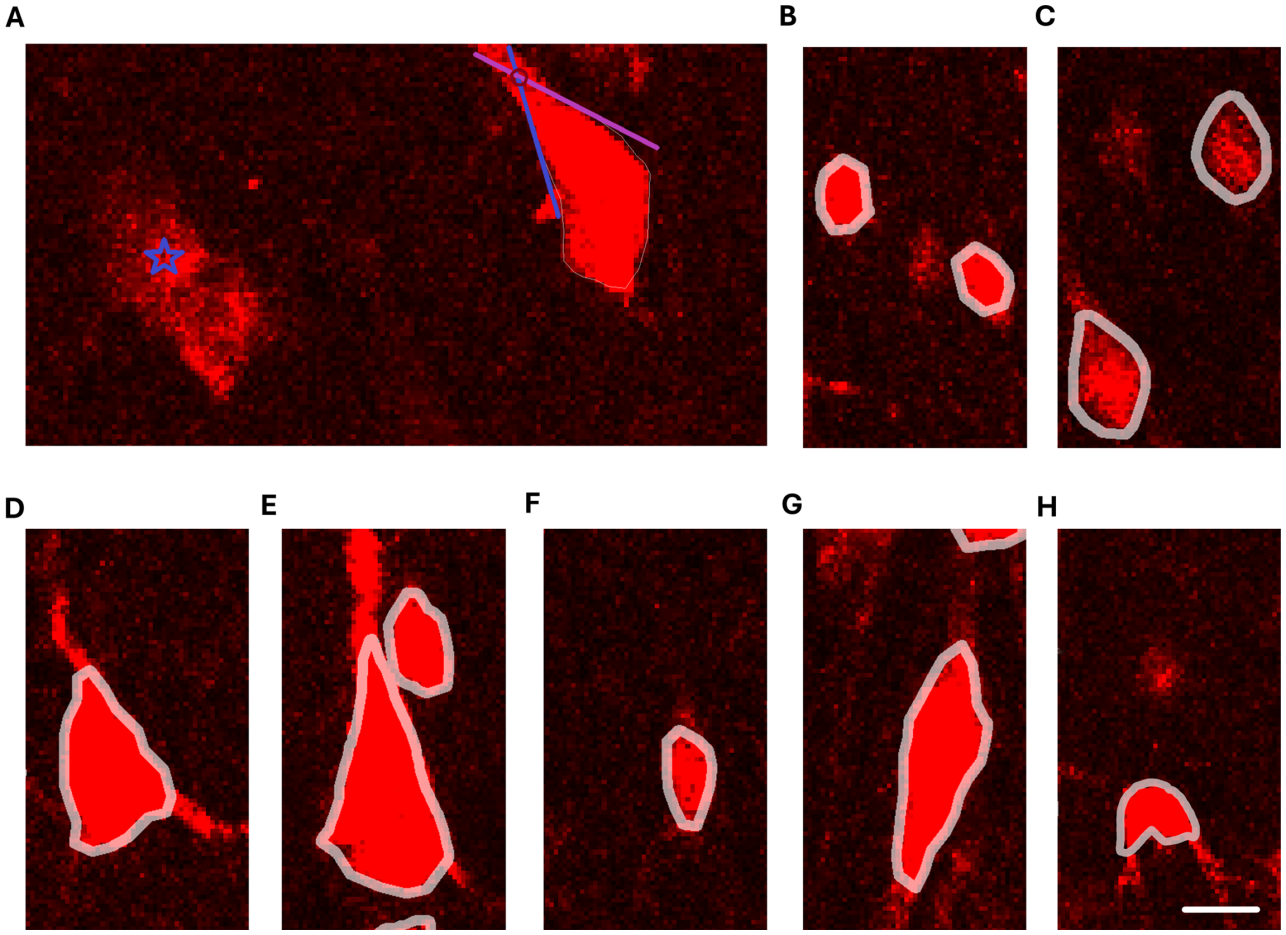
Reconstructions of neuron cell bodies on confocal images of NeuN staining. **(A)** Criteria for delineation of cell body contours. The blue star symbol marks NeuN^+^ staining that is not eligible for reconstruction due to unclear boundaries and poor demarcation between the NeuN^+^ staining and background staining. The pyramidal cell on the right side shows an example of a cell where the boundary between the apical dendrite is and the cell body is not sharp. The blue and purple lines follow the cell body edges adjacent to the apical dendrite and the red circle marks where the extensions of the edges intersect. The intersection denotes the distal boundary of the reconstructed cell body. **(B)** Reconstructions of typical granule cells. **(C)** Reconstructions of small pyramidal cells. **(D)** Reconstruction of a medium pyramidal cell. **(E)** Reconstruction of a large pyramidal cell. **(F)** Reconstruction of a small fusiform cell. **(G)** Reconstruction of a large fusiform cell. **(H)** Reconstruction of a polymorphic cell. Note that the cell body shape of the polymorphic cell is irregular and has indentations in the cell contour. Scale bar: 10 μm.

The neuron cell bodies were reconstructed using Neurolucida 2020 (MBF, Vermont, United States), based on the following principles: (1) the cell body contour was drawn along the very edge of the cell body, i.e., at the border between the cell body signal and the background staining; (2) the cellular processes were not encompassed by the cell body contour; (3) if the transition between the cell body and the cellular process was unclear, the cell body was delineated so that it extended into the cellular process to the point where the extensions of the two adjacent edges of the cell body intersected (Figure 1A).

The reconstructions on each imaged slide were done within a column of cortical tissue. The columns were 2 mm wide at the pial surface and extended towards the border with the white matter, following the vertical orientation of the cells in the tissue. Within the cortical column, each cortical layer (I–VI) was delineated separately. The reconstructions were done by using the *Outline objects* function that automatically detects cell contours in confocal images and produces two-dimensional reconstructions of the cell bodies. The automatically drawn contours were reviewed and corrected by two independent researchers. The cell bodies of a total of 90,723 neurons were reconstructed in this research.

The cell body reconstructions were analyzed using Neurolucida Explorer (MBF, Vermont, USA), which provided the following morphometric parameters for each cell: soma circumference (*Perimeter*), soma surface (*Area*), the largest diameter of the soma contour (*Feret Max*), the smallest diameter of the soma contour (*Feret Min*), ratio between *Feret Max* and *Feret Min* (*Aspect Ratio*), ratio between *Area* and *Feret max* (*Compactness*), degree of indentations of soma circumference (*Convexity*), complexity/irregularity of the soma circumference (*Form Factor*), square of *Compactness* (*Roundness*), and ratio between *Area* and *Convex Area* (*Solidity*) (for a detailed description of each parameter see Supplementary data).

### 2.5 Classification of morphological cell types

In order to be able to create a prediction algorithm for different morphological cell types, 2,850 of the reconstructed cells were manually classified based on their soma shape and size, using the work of von Economo and Koskinas (1925) as reference. Von Economo and Koskinas refer to three main cell types in the cerebral cortex—granule cells, pyramidal cells and fusiform cells. We introduced the category of polymorphic cells to describe cells with irregular and/or unusual shapes that we could not classify into any of these three main cell types. Von Economo and Koskinas further subdivided pyramidal cells by size (using height and width as the main measures) into small, medium, large, and gigantic. Even though they did not further subdivide fusiform cells, the size range they included for this cell type was very large (height ranging from 15 to 30 μm), which is why we classified fusiform cells into the small and large subcategories. It should be noted that the classification proposed by von Economo and Koskinas, and adapted in this study, relies exclusively on the neurons’ soma shape and size. This classification does not take into account the dendritic and axon morphology of the cells.

Therefore, in this research, the cells were classified into the following categories: granule cells, small pyramidal cells, medium pyramidal cells, large pyramidal cells, gigantic pyramidal cells, small spindle cells, large spindle cells, and polymorphic cells (Table 1 and Figures 1B–H).

**Table 1:** Morphological classification of neurons, modified from von Economo and Koskinas (1925).

Cell type	Cell body shape	Average height (μm)	Average width (μm)
Granular	Circular or polygonal	4–8	4–10
Small pyramidal	Triangular	12	10
Medium pyramidal		25	15
Large pyramidal		30–45	15–20
Gigantic pyramidal		>50	>25
Small fusiform	Spindle-shaped	15	10
Large fusiform		30	15
Polymorphic	Does not fit into any other category (irregular shape)	—	—

In order to get a representative sample, manual classification was performed in all three cortical regions in all of the analyzed brains, and the cells were chosen from all cortical layers. It should be noted that, on the slides we analyzed, we found no cells we could reliably manually classify as gigantic pyramidal cells.

Besides the two-dimensional cell body reconstructions utilized for the morphometric analysis, we provided several representative three-dimensional cell body reconstructions (Supplementary material—3D reconstructions).

### 2.6 Quantitative analysis

Quantitative analysis was performed in GraphPad Prism version 10.2.2 (GraphPad Software, La Jolla, United States), DATAtab (2024): Online Statistics Calculator (Graz, Austria) and Orange Data Mining version 3.36.2 (Ljubljana, Slovenia) (Demšar et al., 2013).

For each morphometric parameter, the following descriptive statistics were calculated: mean, standard deviation (SD), 95% confidence interval (CI) for mean, median, interquartile range (IQR), mode, minimum and maximum values, and coefficient of variation (CV).

Neuronal morphometric parameters, similarly to neuron densities (Morales-Gregorio et al., 2023), are typically not normally distributed. This was the case in our study as well—we evaluated the distributions graphically and using the D’Agostino-Pearson omnibus *K*^2^ test, with both methods indicating that the data were likely not sampled from a normal distribution. Therefore, non-parametric tests were more appropriate for our analysis.

The differences in morphometric parameters between cortical regions were compared using the Kruskal–Wallis test with Dunn’s *post hoc* test.

The association between morphometric parameters was assessed using Spearman’s correlation coefficient. The correlation matrix was used to determine which morphometric parameters were most strongly associated with each other (Supplementary Table S2). Since some parameters are mathematically derived from other parameters, we aimed to determine which parameters were most relevant for distinguishing different cell types. We performed factor analysis and assessed the scree plot showing the relationship between the possible factors and the corresponding eigenvalues (Supplementary Figure S2). Based on the eigenvalue criterion (eigenvalue >1), we determined that for our dataset, the optimal number of factors would be three. We then assessed the factor loadings in the rotated component matrix (Supplementary Table S3) as an aid in informing which parameters were most relevant to describing the morphometric characteristics of different cell types.

Receiver operating characteristic (ROC) analysis was used to assess how effectively different cell types could be classified based on different morphometric parameters at varying threshold values. When determining the desired threshold values, we aimed to achieve a high specificity (preferably above 90%) and a reasonable sensitivity (preferably above 70%).

Based on factor analysis and ROC analysis, we concluded that the parameters *Area*, *Aspect Ratio* and *Form Factor* were most useful for differentiating between different morphological cell types (for a detailed description see Supplementary data).

t-distributed stochastic neighbor embedding (t-SNE) was used to visualize the morphometric data by plotting each datapoint on a two-dimensional map. The t-SNE was run with only the morphometric parameters and two categorical variables (cortical layer and cortical regions) in order to assess whether the grouping of cortical layers would be similar to the results obtained with the neural network prediction algorithm.

The neural network widget in Orange was used to predict which morphological cell type each reconstruction belonged to, based on the cell’s morphometric parameters. The neural network widget in Orange uses the multi-layer perceptron supervised learning algorithm developed by scikit-learn (Orange Data Mining, n.d.; scikit-learn, n.d.). The manually classified reconstructions were used as the training data set, while the rest of the reconstructions (87,873 reconstructed cells) constituted the test data set. The predictions from the test data set were used to determine the relative proportions of each morphological cell type. For this analysis, we excluded cells that the prediction algorithm classified into a respective category with less than 50% certainty. The total number of excluded cells was 6026, i.e., 6.86% of the total number of cells in the test data set (Supplementary Table S4).

The chi-square test was used to compare the morphological composition (derived from the predictions of morphological cell types) of cortical regions.

The proportions between the three major morphological cell types (granule, pyramidal and fusiform cells) were visualized using ternary diagrams. The proportions between all seven morphological cell types were visualized using radar diagrams.

For all statistical tests, *p* < 0.05 was considered statistically significant.

## 3 Results

**Figure 2:**
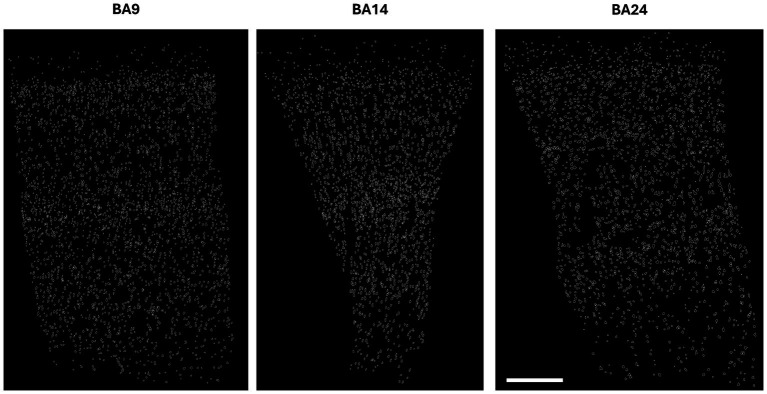
Reconstructions of cortical columns of Brodmann area 9, 14r and 24. Scale bar: 500 μm.

Our analysis of the cell body reconstructions revealed that the cytoarchitectonic descriptions of BA9, BA14r and BA24 were reflected in the morphometric measures of the cells. Our qualitative observations of the reconstructed cortical columns were also in line with the qualitative descriptions of the analyzed cortical regions (Figure 2; Supplementary Figure S3).

### 3.1 Average cell size and elongation differed significantly between cortical regions

**Figure 3:**
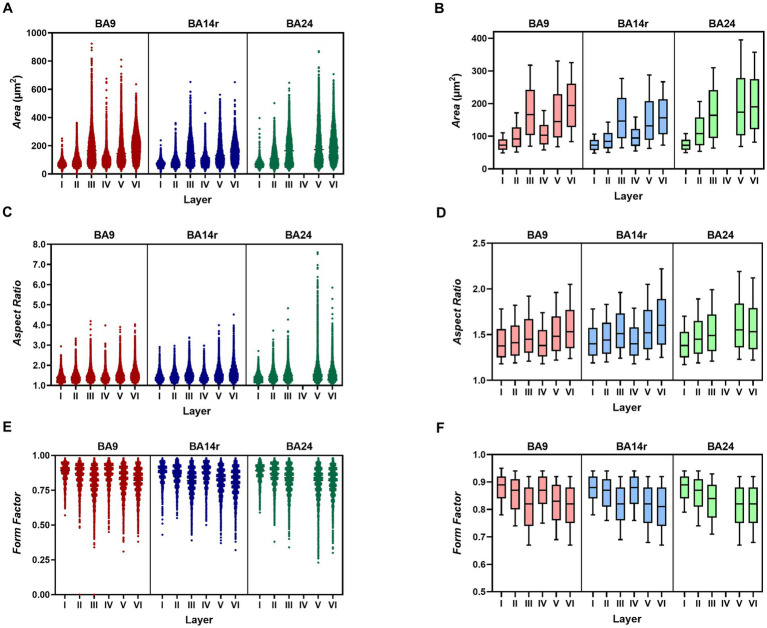
Values of morphometric parameters in each layer of each cortical region. **(A)** Scatterplot showing the *Area* values for individual cells. Note the increase in cell size in layer III of BA9 and layer V of BA24. **(B)** Box-plot showing the median *Area* values. The upper and lower borders of the rectangle represent the third and first quartile respectively, while the error bars denote the 90th and 10th percentiles. Note the large median cell sizes in layers III, V and VI, compared to layers I, II and IV. **(C)** Scatterplot showing *Aspect Ratio* values. Note the substantial increase in cell elongation in layer V of BA24. **(D)** Box-plot showing *Aspect Ratio* values. Note the gradual increase in cell elongation from BA9, through BA14r to BA24. **(E)** Scatterplot showing Form Factor values. Note that the lowest individual values are found in BA24. **(F)** Box-plot showing Form Factor values. Note the lower values in layers III, V and VI compared to layers I, II, and IV.

The average cell size, measured by the parameter *Area*, differed significantly between all three cortical regions (*p* < 0.001 for all comparisons, Kruskal–Wallis test). The cells in BA14r were the smallest on average, with the lowest average *Area* values (140.53 ± 80.37 μm^2^). Although the cells in BA24 had the highest average *Area* values (176.55 ± 108.83 μm^2^, compared to 164.93 ± 98.85 μm^2^ in BA9), the largest individual cells were found in BA9 with *Area* values up to 923.41 μm^2^ (Figure 3 and Table 2).

**Table 2:** Descriptive statistics for morphometric parameters for the three analyzed cortical regions.

	BA	Mean ± SD	95% CI for mean	CV	Median	IQR	Mode	Minimum	Maximum
*Area* (μm^2^)	9	164.93 ± 98.85	163.93–165.93	59.94%	139.66	131.82	51.76	12.19	923.41
	14	140.53 ± 80.37	139.59–141.46	57.19%	117.53	107.33	52.72	13.09	652.10
	24	176.55 ± 108.83	175.20–177.90	61.64%	151.03	148.89	61.32	15.35	869.85
*Aspect Ratio*	9	1.52 ± 0.31	1.52–1.53	20.36%	1.45	0.37	1.31	1.04	4.19
	14	1.56 ± 0.33	1.56–1.57	20.86%	1.5	0.39	1.34	1.04	4.52
	24	1.59 ± 0.41	1.58–1.59	26.09%	1.5	0.42	1.36	1.05	7.60
*Form Factor*	9	0.82 ± 0.09	0.82–0.82	11.48%	0.84	0.12	0.88	0.31	0.98
	14	0.82 ± 0.09	0.82–0.83	10.83%	0.84	0.12	0.89	0.32	0.98
	24	0.82 ± 0.10	0.82–0.82	11.56%	0.84	0.13	0.88	0.23	0.98

The average elongation of the cells, measured by the parameter *Aspect Ratio*, was significantly lower in BA9 (1.52 ± 0.31) compared to BA14r and BA24 (*p* < 0.001), while the difference between BA14r and BA24 was not significant (*p* = 0.111), though cells in BA24 were slightly more elongated on average (1.59 ± 0.41) than cells in BA14r (1.56 ± 0.33). It is also worth noting that the individual *Aspect Ratio* values were by far the highest in BA24, with maximum values up to 7.60 (compared to 4.19 and 4.52 in BA9 and BA14r respectively). In addition, the CV for *Aspect Ratio* was substantially higher in BA24 (26.09%) than in BA9 (20.36%) and BA14r (20.86%), indicating a higher diversity in cell elongation in this region (Figure 3 and Table 2).

The irregularity and complexity of the cell shape, measured by the parameter *Form Factor*, did not significantly differ between the three cortical regions. Individual cells with the highest cell shape irregularity were found in BA24 with minimum Form Factor values reaching 0.23, compared to 0.31 in BA9 and 0.32 in BA14r (Figure 3 and Table 2).

### 3.2 Morphometric parameters differed significantly between cortical layers

The values of *Area*, *Aspect ratio* and *Form Factor* differed significantly between all cortical layers (*p* < 0.001 for all comparisons, Kruskal–Wallis test).

The average cell sizes were substantially lower in layers I, II and IV than in layers III, V and VI in all three of the analyzed cortical regions. The largest individual cells were found in layer III of BA9. The CV for *Area* was highest in layer V in all cortical regions, indicating that layer V had the highest diversity in cell size (Figure 3 and Supplementary Table S5).

On average, cells were far less elongated in layers I, II and IV than in layers III, V and VI in all cortical regions. The most elongated individual cells were found in layer V of BA24, and this layer in BA24 also had by far the highest CV (33.55%) for *Aspect Ratio* (Figure 3 and Supplementary Table S5).

On average, cells had the most irregular shape in layers III, V and VI in all cortical regions (Figure 3 and Supplementary Table S5). These layers also had a higher CV for *Form Factor*, indicating a higher diversity in cell shape complexity compared to layers I, II and IV.

### 3.3 Neurons can be classified into morphological cell types based on *Area*, *Aspect Ratio* and *Form Factor*

**Figure 4:**
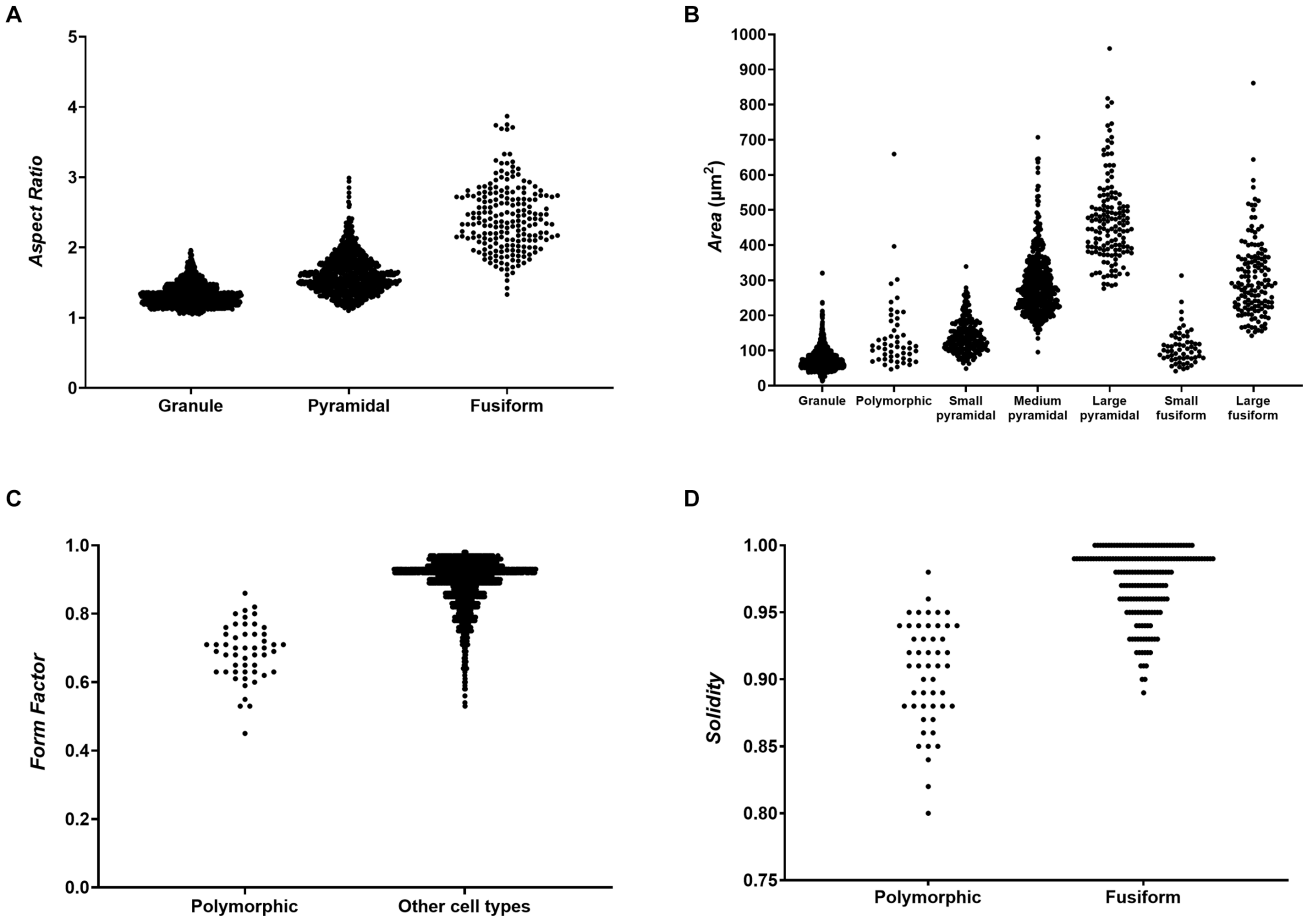
Scatter plots showing values of morphometric parameters for different morphological cells types. **(A)** *Aspect Ratio* values for granule, pyramidal and fusiform cells showing that the three main morphological cell types can be distinguished based on cell elongation. **(B)** *Area* values for all cell types showing that subtypes of pyramidal and fusiform cells can be distinguished based on cell size. **(C)** *Form Factor* values for polymorphic cells and all other cell types showing that polymorphic cells have, on average, more complex cell contours. **(D)** *Solidity* values for polymorphic and fusiform cells showing that polymorphic cells have more indentations in their cell contours, which enables them to be distinguished from fusiform cells with similar *Form Factor* values.

Based on Factor and ROC analysis of the manually classified reconstructions, we determined that the three major morphological cell types—granule cells, pyramidal cells and fusiform cells—could be best distinguished using the parameter *Aspect Ratio* (Figure 4). This is due to the fact that granule cells were the least elongated cell type, while fusiform cells were the most elongated, with pyramidal cells typically falling in between these two categories. ROC analysis revealed that pyramidal and fusiform cells could reliably be distinguished by choosing a threshold for *Aspect Ratio* values of 1.86 (specificity: 90.29%, sensitivity: 82.55%). Distinguishing between pyramidal and granule cells based on *Aspect Ratio* was more challenging, and we determined an acceptable threshold value to be 1.38 (specificity: 80.94%, sensitivity: 69.65%) (Table 3).

**Table 3:** Threshold values of morphometric parameters for distinguishing morphological cell types.

Cell type	Proposed threshold values	
Granule	*Aspect Ratio* < 1.38	
Small pyramidal	*Aspect Ratio*: 1.38–1.86	*Area* < 202.2 μm^2^
Medium pyramidal		*Area*: 202.2–394.5 μm^2^
Large pyramidal		*Area* > 394.5 μm^2^
Small fusiform	*Aspect Ratio* > 1.86	*Area* < 161.2 μm^2^
Large fusiform		*Area* > 161.2 μm^2^
Polymorphic	*Form Factor* < 0.79 *Solidity* < 0.95	

The subtypes of pyramidal and fusiform cells could most easily be distinguished using the parameter *Area* (Figure 4). Small and large fusiform cells could be distinguished very reliably with a threshold value of 161.2 μm^2^ for *Area* (specificity: 96.58%, sensitivity: 90.00%). Small and medium pyramidal cells could also be reliably distinguished with a threshold value of 202.2 μm^2^ (specificity: 90.38%, sensitivity: 89.17%). Distinguishing between medium and large pyramidal cells based on *Area* was more challenging, but we determined an acceptable threshold value to be 394.5 μm^2^ (specificity: 89.17%, sensitivity: 70.39%) (Table 3).

Polymorphic cells could be best distinguished using the parameter *Form Factor* (Figure 4), due to such cells having irregular cell body shapes with a high circumference complexity (i.e., lower *Form Factor*). We determined an acceptable threshold value for *Form Factor* to be 0.79 (specificity: 90.20%, sensitivity: 71.32%). A more detailed analysis revealed that distinguishing polymorphic from fusiform cells based only on *Form Factor* values was not sufficiently reliable (the threshold of 0.79 for these two cell types had only 11.05% specificity). Therefore, we analysed other morphometric parameters and determined that polymorphic and fusiform cells could be distinguished by analysing the parameter *Solidity*, since polymorphic cells had more indentations in their cell contours, and thus lower *Solidity* than fusiform cells. An acceptable threshold value for *Solidity* was 0.95 (specificity: 86.00%, sensitivity: 83.87%) (Table 3).

The descriptive statistics for the different cell types are shown in Supplementary Table S6.

### 3.4 Neural network predictions revealed the morphological composition of cortical regions and layers

After manual classification, the rest of the reconstructions were classified using a neural network prediction algorithm and the predictions were used to calculate the relative proportions of morphological cell types for each cortical region and layer.

Our analysis determined that granule cells were the predominant cell type in BA14r (47.48% of all cells), while the relative proportions of granule cells were a bit lower in BA9 (41.76%) and BA24 (39.60%). Pyramidal cells were more prevalent in BA9 (46.39%) than in BA14r (43.33%) and BA24 (41.03%). Fusiform and polymorphic cells were most prevalent in BA24 (9.63 and 9.74% respectively), whereas these two cell types combined constituted only around 10% of cells in BA9 and BA14r. The differences in the morphologic composition between cortical regions were statistically significant (*p* < 0.001, chi-squared test).

**Figure 5:**
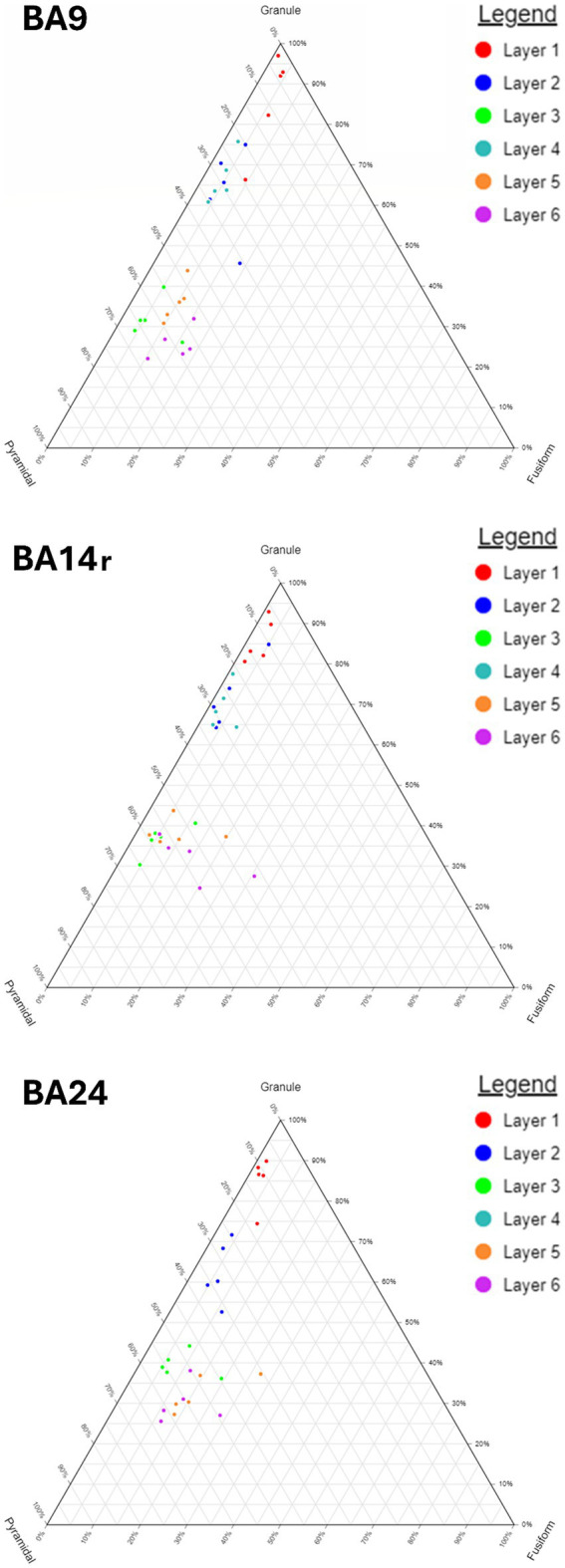
Ternary diagrams showing the relative proportions of granule, pyramidal and fusiform cells in Brodmann areas 9, 14r and 24. Note the gradual increase in the proportion of fusiform cells from BA9 through BA14r to BA24. Also note the relatively similar composition of layers II and IV as well as the difference in their composition to layers III, V, and VI.

**Figure 6:**
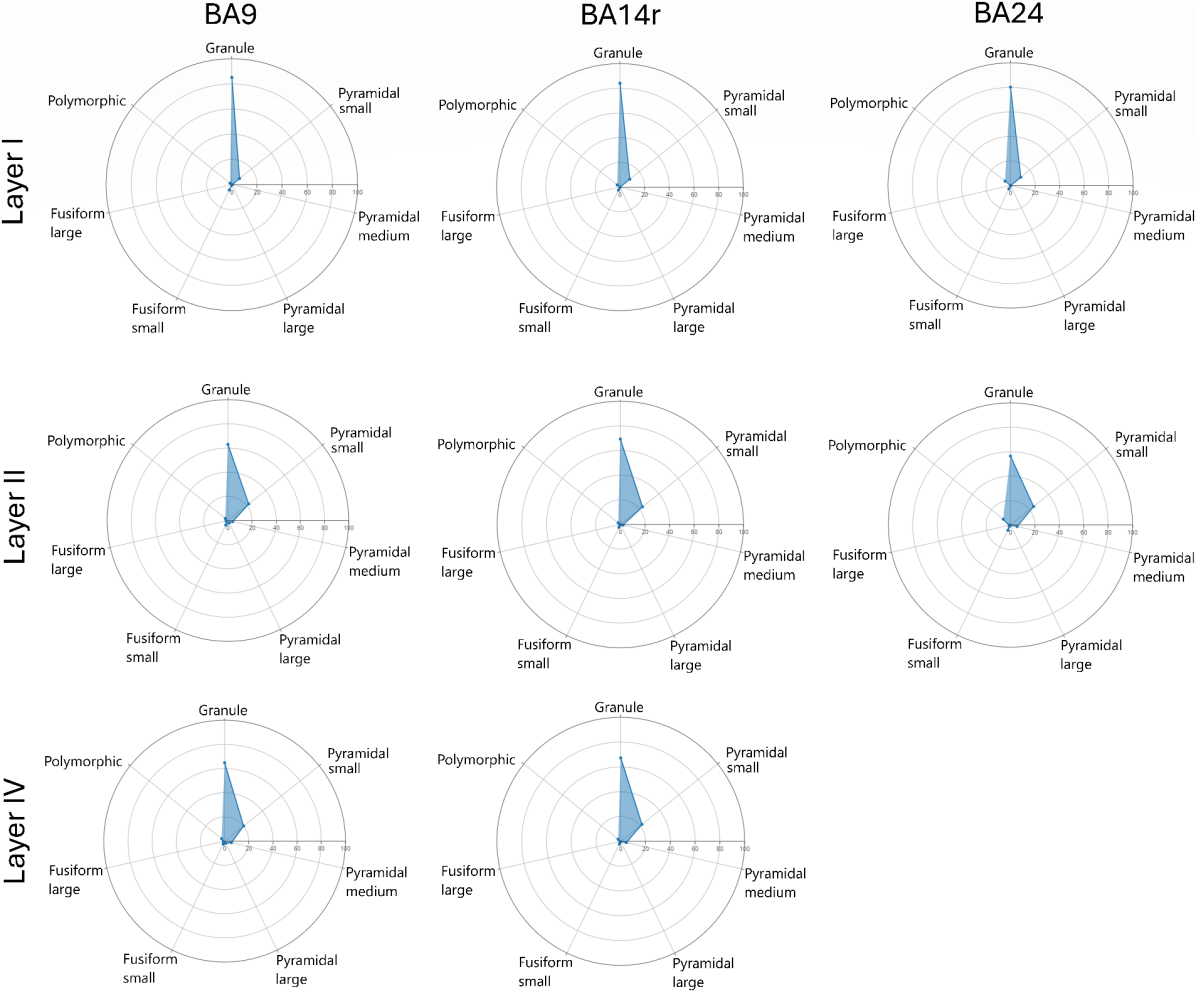
Radar diagrams showing the morphological composition of layers I, II and IV in Brodmann areas 9, 14r and 24. Note the similar compositions of layers II and IV as well as the predominance of granule cells in layer I. Also note the slight increase in the proportion of polymorphic cells in BA24.

**Figure 7:**
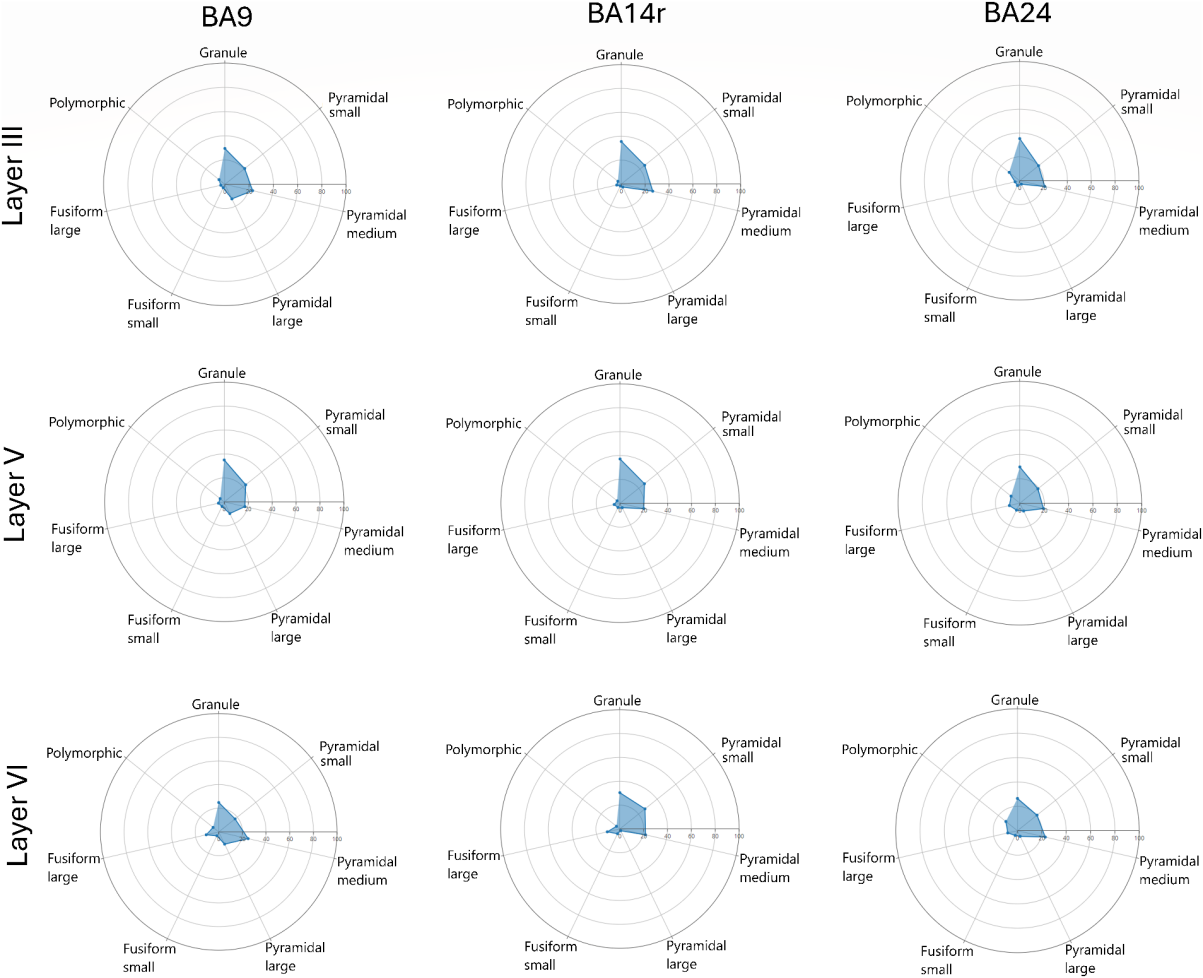
Radar diagrams showing the morphological composition of layers III, V and VI in Brodmann areas 9, 14r and 24. Note the increase in the proportion of large pyramidal cells in layer III of BA9 as well as the increase in the proportion of cells classified as large fusiform in layer V of BA24. Also note the slight increase in the proportion of polymorphic cells in BA24.

When analyzing individual cortical layers, it was apparent that layers I, II and IV had very a different morphological structure compared to layers III, V and VI, in all three regions (Figures 5–7).

In layer I, granule cells constituted over 80% of all cell types in all three cortical regions, with the prediction algorithm classifying the rest of the cells as either small pyramidal, small fusiform or polymorphic. No cells in layer I were classified as medium or large pyramidal or as large fusiform.

In layer II, granule cells constituted over 65% of all cell types in BA9 and BA14r, and over 58% in BA24, with most of the other cells (above 20% in each region) being classified as small pyramidal. Less than 1% of cells in this layer were classified as large pyramidal or large fusiform.

In layer III, pyramidal cells were the most prevalent cell type, constituting above 50% of all cells in BA9 and BA14r, and 43.80% in BA24. The prediction algorithm classified only 4.98 and 7.01% of the pyramidal cells as large pyramidal in BA14r and BA24 respectively, while in BA9 23.07% of pyramidal cells were classified as large pyramidal. Granule cells were the second most common cell type in this layer, and were slightly more prevalent in BA14r (38.12%) and BA24 (37.77%) than in BA9 (34.64%). Fusiform and polymorphic cells were relatively rare in BA9 and BA14r (5% or lower in both regions for both cell types), but were more prevalent in BA24 (fusiform cells—7.68%, polymorphic cells—10.75%).

In layer IV, granule cells were once again the most prevalent cell type and constituted more than 65% of all cell types in BA9 and BA14r, similar to layer II in these regions. Also similar to layer II, the second most prevalent cell type were small pyramidal cells (around 20% in both regions). However, the number of cells classified as medium and large pyramidal in layer IV was slightly larger than in layer II in both cortical regions.

In layer V, pyramidal cells were the most common type (above 45% in all three cortical regions), followed by granule cells (above 35% in all cortical regions). Compared to layer III, almost twice as many cells were classified as fusiform cells in all three cortical regions, with the proportion of cells classified as fusiform cells in BA24 being especially high (13.74%). In addition, more fusiform cells in layer V were classified as large than as small, which is in contrast to layers I–IV.

In layer VI, pyramidal cells were also the most common cell type (above 45% in all three cortical regions), and granule cells were the second most common, though a bit less prevalent than in layer V (around 30% in all cortical regions). In BA9 and BA14r, almost twice as many cells were classified as fusiform cells than in layer V (above 13% in both regions). This was not the case in BA24, where the proportion of fusiform cells was similar in layers V (13.74%) and VI (11.64%). Out of all the fusiform cells in layer VI, above two thirds were classified as large fusiform in all three cortical regions. In addition, layer VI had the largest proportion of polymorphic cells compared to the other layers in all three cortical regions.

The morphological composition of layers I, II and IV differed only slightly between cortical regions, however, layers III, V and VI revealed relatively region-specific morphological compositions as seen on the radar diagrams (Figures 6, 7).

**Figure 8:**
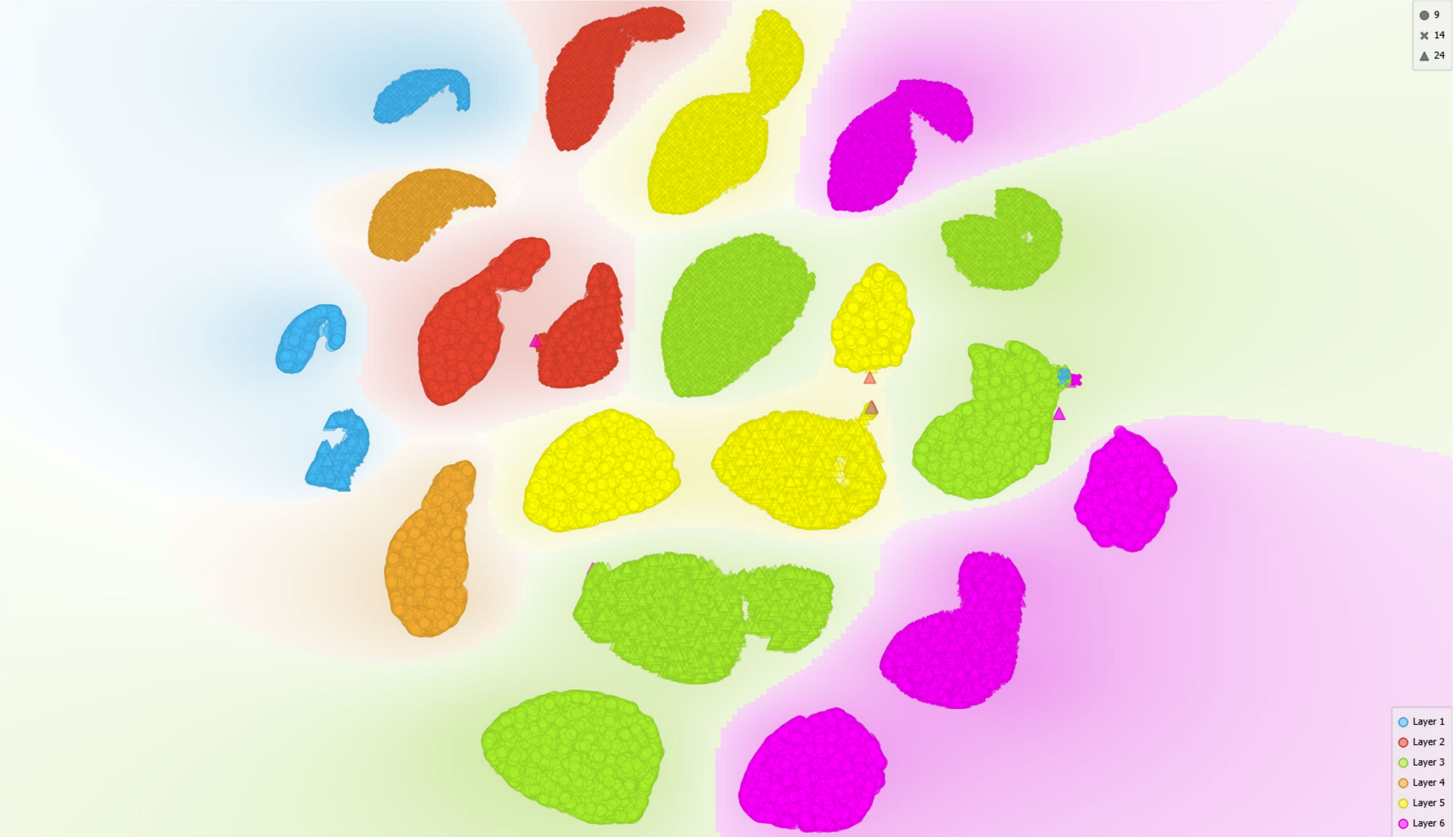
Results of t-SNE plotted on a two-dimensional diagram. Note the lack of clear separation in the grouping of layers II and IV as well as layers III, V, and VI.

The results of t-SNE were consistent with the results we obtained by analyzing the neural network predictions (Figure 8). Cells from layers I, II and IV were grouped closer together than cells from layers III, V and VI. No clear separation in the grouping of cells from layers II and IV could be observed, which is reflected in the similar morphological composition of these layers, and the same was true for layers III, V and VI. It should be noted that the groupings of cortical layers by t-SNE were derived independently from the morphological compositions determined by the neural network.

## 4 Discussion

In this research we reconstructed the cell bodies of NeuN^+^ cells in three cortical regions of the PFC—BA9, BA14r and BA24. We analyzed the morphometric characteristics of these cell bodies and compared them between these cortical regions and all cortical layers, finding significant differences in the values of *Area*, *Aspect Ratio* and *Form Factor*. We manually classified a portion of the reconstructions into morphological cell types. We used ROC analysis to determine the best thresholds for distinguishing between cell types based on morphometric parameters. We also used the manually classified reconstructions as training data for a neural network prediction algorithm that was then used to classify the rest of the reconstructions. Based on the neural network classification, we established the morphological composition of cortical layers in the three analyzed cortical regions and determined the specificities of each region and layer.

### 4.1 BA9 was characterized by an abundance of large pyramidal cells present in layer III

Individual neurons in BA9 were the largest (highest *Area* values) among the three cortical regions, though the average neuron size was somewhat higher in BA24. Laminar analysis showed that such large cells were found almost exclusively in layer III of BA9. This seemed to indicate an abundance of large pyramidal cells in the deep part of layer III, which is characteristic for homotypical isocortical regions in the PFC that have extensive associative projections (Petanjek et al., 2019). This relative abundance of large pyramidal cells in layer III of BA9 was confirmed using the neural network prediction model, with BA9 having over thrice as many large pyramidal cells in layer III than BA14r and BA24.

Neurons in BA9 were less elongated compared to BA14r and BA24. This is in concordance with the cytoarchitectonic descriptions of Brodmann (1909) and von Economo and Koskinas (1925), who described a high proportion of granule and pyramidal cells in this region. This was also in line with the results of our neural network prediction model, which showed the lowest proportion of fusiform cells in this region.

### 4.2 BA14r was characterized by smaller average cell size and a relative abundance of granule cells in layer III

Neurons in BA14r were, on average, the smallest among the analyzed cortical regions, which was reflected in the relatively low proportions cells classified as large pyramidal and large fusiform by the prediction model. Cytoarchitectonic descriptions of this region mention a significant decrease in the proportion of large pyramidal cells (von Economo and Koskinas, 1925; Ongür et al., 2003), which is consistent with our finding. *Aspect Ratio* values of cells in this region were higher than in BA9, which is in line with the pattern of cell elongation from dorsal to orbital regions of the PFC (von Economo and Koskinas, 1925; Ongür et al., 2003). However, since the overall proportion of fusiform cells did not increase in BA14r compared to BA9, the slightly higher cell elongation in this region cannot be explained by a change in the prevalence of fusiform morphological cell types. It is likely that this increase in cell elongation is a consequence of morphological modifications of pyramidal cells. This process of pyramidal cell transformation into elongated modified pyramidal cells was described by von Economo and Koskinas (1925) as spindle transformation (*Verspindelung*), and was described predominantly in layers III and V. These descriptions are consistent with our findings, because we determined the largest increase in cell elongation in layers III, V and VI of BA14r compared to BA9.

Interestingly, even though BA14r is usually described as dysgranular, the proportion of granule cells was generally high in this region. In particular, the proportion of granule cells in layer III of BA14 was slightly higher than in BA9. A likely explanation for this finding is the more difficult delineation between layers III and IV, because of which more granule cells seem to be present in layer III.

### 4.3 BA24 was characterized by extreme cell elongation in layer V

Neurons in BA24 were the largest on average among the three cortical regions. The average increase in cell size was probably, at least partly, due to the general increase in relative thickness of layers V and VI in BA24, since these layers contained, on average, the largest neurons in general. In addition, the average cell size in layer V of BA24 was exceptionally high with the largest individual cells also being found in layer V of this region. This is in contrast to BA9 and BA14r, where the largest individual cells were found in layer III.

Cell elongation in BA24 was higher on average than in BA9 and BA14r. Further analysis showed that this increase in cell elongation was most prominent in layer V of BA24. This was further confirmed by the neural network prediction model, which showed an increase in the proportion of large fusiform cells in this region, particularly in layer V. This increase is likely due to the presence of specialized cells, called von Economo neurons (VENs) (von Economo and Koskinas, 1925; von Economo, 1927; Banovac et al., 2021; Petanjek et al., 2023). VENs are large, highly elongated rod-shaped cells abundant only in layer V of two cortical regions in humans—the anterior cingulate cortex (which corresponds to BA24) and the fronto-insular cortex.

The increase in cell size, as well as the increase in cell elongation in layer V of BA24 is highly indicative of the presence of VENs. Since in the training data we did not manually classify any specialized cell types, it is most likely that the prediction algorithm classified VENs as large fusiform cells, since that was the cell type they were most similar to. However, it should be noted that VENs are specialized cells and should be distinguished from the typical fusiform cells commonly found in the deep layers of the cerebral cortex.

Interestingly, neurons in BA24 had the lowest individual minimum values of *Form Factor*, indicating that this region contained more cells with highly irregular cell shapes and complex cell contours. This is in concordance with the results of the neural network prediction model, which showed a substantially higher proportion of polymorphic and fusiform cells in this region.

### 4.4 Objective delineation of cortical regions and layers may have translational potential

Determining the distribution of different neuron types in the cerebral cortex enables the objectivization of morphological and functional organization within specific cortical regions and layers. The results of our research could help develop a standardized way of identifying cortical regions and layers without relying on experts’ subjective assessment. This research also has translational potential, with possible applicability in the objective detection of morphological alterations of neural microcircuitry in neuropathological conditions. For example, such an algorithm could be further developed to assist in the evaluation of the histological characteristics of CNS tumors, or in the assessment of progression of various neurodegenerative diseases.

The results of the neural network prediction model were in line with our expectations based on qualitative descriptions of the examined regions’ cytoarchitectonics. This supports the notion that the prediction model was relatively successful in classifying cells based on the training data, and suggests that such models could be utilized in aiding morphological analyses in neuroscience research. Future research should focus on cross-validation of the model, as well as on applying such prediction models to other cortical regions, neuropathological conditions, and other species besides humans. When using such an algorithm in other cortical regions, it might be necessary to take into account the presence of additional cell types—e.g., Betz cells in the primary motor cortex or Purkinje cells in the cerebellar cortex.

A conceivable end-goal should be producing a quality model, utilizing machine learning, that could accurately delineate cortical regions and layers, as well as determine their morphological compositions and evaluate possible morphological alterations in neuropathology. In conclusion, we demonstrated that supervised machine learning, if provided with sufficient quality input data, could significantly aid in defining the morphological characteristics of the cerebral cortex.

## Data availability statement

The original contributions presented in the study are included in the article/Supplementary material, further inquiries can be directed to the corresponding author.

## Ethics statement

The studies involving humans were approved by University of Zagreb School of Medicine Ethics Committee. The studies were conducted in accordance with the local legislation and institutional requirements. The human samples used in this study were acquired as part of a previous study for which ethical approval was obtained. Written informed consent for participation was not required from the participants or the participants’ legal guardians/next of kin in accordance with the national legislation and institutional requirements.

## Author contributions

MP: Data curation, Formal analysis, Investigation, Methodology, Resources, Software, Validation, Visualization, Writing – original draft. ZP: Conceptualization, Funding acquisition, Resources, Supervision, Writing – review & editing. IB: Conceptualization, Formal analysis, Funding acquisition, Investigation, Methodology, Project administration, Resources, Software, Supervision, Validation, Visualization, Writing – original draft.
