# Supplementary material for: A novel approach to cytoarchitectonics: developing an objective framework for the morphological analysis of the cerebral cortex
Source: Front Neuroanat. 2024 Aug 12;18:1441645. doi: 10.3389/fnana.2024.1441645 (PMC11345133; doi:10.3389/fnana.2024.1441645)
Supplement: Supplementary file 1 [file Data_Sheet_1.docx]

Supplementary Material

# Supplementary Data

## Delineation of cortical regions and layers

In this research, we used the work of von Economo and Koskinas (1925) as our main reference for delineating cortical regions and layers, since their description of the cytoarchitectonics of the human cerebral cortex is by far the most comprehensive and detailed. However, since the nomenclature developed by von Economo and Koskinas is not as widely used, we cross-referenced their descriptions with those of Brodmann (1909), Petrides and Pandya (1999), Vogt et al. (1995), and Ongür and Price (2000) in order to ensure consistency with the more common nomenclature.

For initial confirmation of the cytoarchitectonic features of the cortical regions, we used Nissl staining, since the classical cytoarchitectonic works were done using this staining as well. We used NeuN staining for the morphometric reconstructions and subsequent analyses for several reasons: 1) NeuN as a marker visualizes only neurons, so we could be sure we were evaluating only neuron morphology, 2) the automatic reconstruction of neuron cell bodies was, in our case, more precise on fluorescent NeuN staining, 3) layer IV is usually more easily delineated on NeuN staining.

For the Nissl staining we used the following protocol: the sections were treated with 0.5% Cresyl violet (Sigma, USA)) solution for 3 min, followed by differentiation in acetic acid in 70% ethanol for 5 s, and a series of 96% and 100% ethanol until the staining was optimal.

### Brodmann area 9

Von Economo and Koskinas describe the granular frontal area FD as the region located on “the superolateral convexity of the rostral one-third of the frontal lobe” (for a detailed anatomical localization see Figure 1 from von Economo and Triarhou (2009)). Based on its localization and its cytoarchitectonic description, this region corresponds to BA9 in Brodmann’s map. In addition, BA4 best corresponds to the precentral area FA, BA6 to the agranular frontal area FB, and BA8 to the intermediate frontal area FC.

It should be noted that von Economo and Koskinas also described several modifications of area FD in the middle and inferior frontal gyri (e.g. FDΔ and FDΓ), however, we always sampled the images we analyzed from the dome of the superior frontal gyrus at the part where the cytoarchitectonics of this region should be most typical according to the depiction by von Economo and Koskinas.

Von Economo and Koskinas described the cytoarchitectonic features of this area as follows: 1) layer I is thin, but slightly more cellular than in surrounding areas of the frontal cortex; 2) layer II is distinct, contains granule cells and small pyramidal cells, the border with layer III is not completely clear; 3) layer III contains pyramidal cells that are smaller than in BA4, 6 and 8, but large pyramidal cells are present in the deep parts of layer III; 4) layer IV is easily delineated from layer III and V, it contains small cells, round and triangular in shape; 5) layer V is clearly subdivided into a cell-dense sublayer Va, and a cell-poor sublayer Vb; 6) layer VI is also clearly subdivided into sublayers VIa and VIb; it can relatively easily be delineated from layer V and the white matter, and contains common fusiform cells typical for this layer.

Petrides and Pandya (1999) add the following in their description of BA9: 1) layer II is compact; 2) layer III contains “a number of large, deeply stained pyramidal neurons”; 3) layer IV is relatively narrow compared to surrounding cortical regions (BA46 and BA9/46).

### Brodmann area 14r

Brodmann initially did not describe an area 14 in humans, only in macaque monkeys. However, Ongur and Price found an analogy with the monkey area 14 in the human straight gyrus (*gyrus rectus*) (see Figure 4 in Ongür and Price, 2000, and Figure 2 in Ongür et al., 2003). They described BA14 as lying within the dome of the straight gyrus – its rostral portion (BA14r) has a poorly delineated layer IV (dysgranular cortex), while its caudal portion (BA14c) lacks a defined layer IV (agranular cortex) as well as subdivision of layer V.

Von Economo and Koskinas described this region as the internal area recta modification (area FG_i_) and it was characterized by layer V becoming relatively thicker at the dome of the gyrus than at the walls, with layer III becoming thinner at the dome. They also state the following features of this area: 1) relatively small cells compared to other areas of the frontal lobe; 2) less developed layer IV; 3) layer V is relatively thick, subdivided into sublayers Va and Vb, and most clearly delineated out of all the layers; 4) layer VI is not well demarked from the white matter at the dome of the gyrus.

### Brodmann area 24

Von Economo and Koskinas described the anterior cingulate cortex (ACC) as the agranular anterior limbic area LA, with three modifications: LA_1_ (“covering the dorsal and external wall of the cingulate gyrus”), LA_2_ (“covering the dome of the cingulate gyrus”), and LA_3_ (“covering the internal wall of the cingulate gyrus”). This region corresponds to BA24. We always sampled the images we analyzed from the dome of the cingulate gyrus (area LA_2_).

Von Economo and Koskinas described the cytoarchitectonic features of this area as follows: 1) layer I is relatively thick, especially in LA_2_; 2) layer II is not well developed, contains small granule and triangular cells and is difficult to delineate from layer III; 3) contains small and medium pyramidal cells, while large pyramidal cells are scarce or not present; 4) layer IV is absent, except at the borders with adjacent granular areas of the frontal lobe; 5) layer V is very thick in LA_1_ and LA_2_, and is clearly subdivided into sublayers Va and Vb; sublayer Vb in LA_2_ contains so-called “rod or corkscrew cells” – extremely elongated specialize cells, which we nowadays call von Economo neurons (VENs); 6) layer VI is also relatively thick, is clearly subdivided into sublayers VIa and VIb, and contains fusiform cells. In general, this region is characterized by the predominance of layers V and VI compared to layers I – III, with layers V and VI amounting up to two-thirds of the total cortical thickness.

Vogt et al. (1995) describe a division of BA24 into areas 24a, 24b, 24c, mostly corresponding to the subdivision of area LA by von Economo and Koskinas. Using their terminology, area LA_2_ corresponds most to BA24b.

## Morphometric analysis

The analyzed morphometric parameters are defined in Neurolucida Explorer as follows:

1. *Perimeter* – length of the contour representing the cell body,
2. *Area* – the two-dimensional cross-sectional surface contained within the boundary of the cell body,
3. *Feret Max* – the largest dimension of the cell body contour,
4. *Feret Min* – the smallest dimension of the cell body contour,
5. *Aspect Ratio* – calculated as: $\frac{Feret max}{Feret min}$, indicates the degree of cell body elongation, with a value of 1 indicating a completely flat cell body (i.e. a circle) and higher values indicating more elongated cell bodies,
6. *Compactness* – calculated as:$\frac{\sqrt{\frac{4}{\pi} \times Area}}{Feret max}$ ; values range from 0 to 1, with 1 being the most compact cell body shape (i.e. a circle),
7. *Convexity* – calculated as: $\frac{Convex Perimeter}{Perimeter}$; values range from 0 to 1, with 1 being a cell body with no indentations (e.g. a circle, ellipse or square),
8. *Form Factor* – calculated as: $\frac{4\pi\times Area}{{Perimeter}^{2}}$; values range from 0 to 1, indicates the complexity of the *Perimeter* of the cell body; cells with more irregular contours have lower *From Factor* values,
9. *Roundness* – calculated as: ${Compactness}^{2}$; values range from 0 to 1, used to better differentiate between cell bodies with similar *Compactness* values,
10. *Solidity* – calculated as: $\frac{Area}{Convex Area}$; values range from 0 to 1, indicates the area enclosed by a “rubber band” stretched around a contour (*Convex Area*); indentations in the contour decrease the *Solidity* of the cell body.

# Supplementary Figures and Tables

## Supplementary Figures


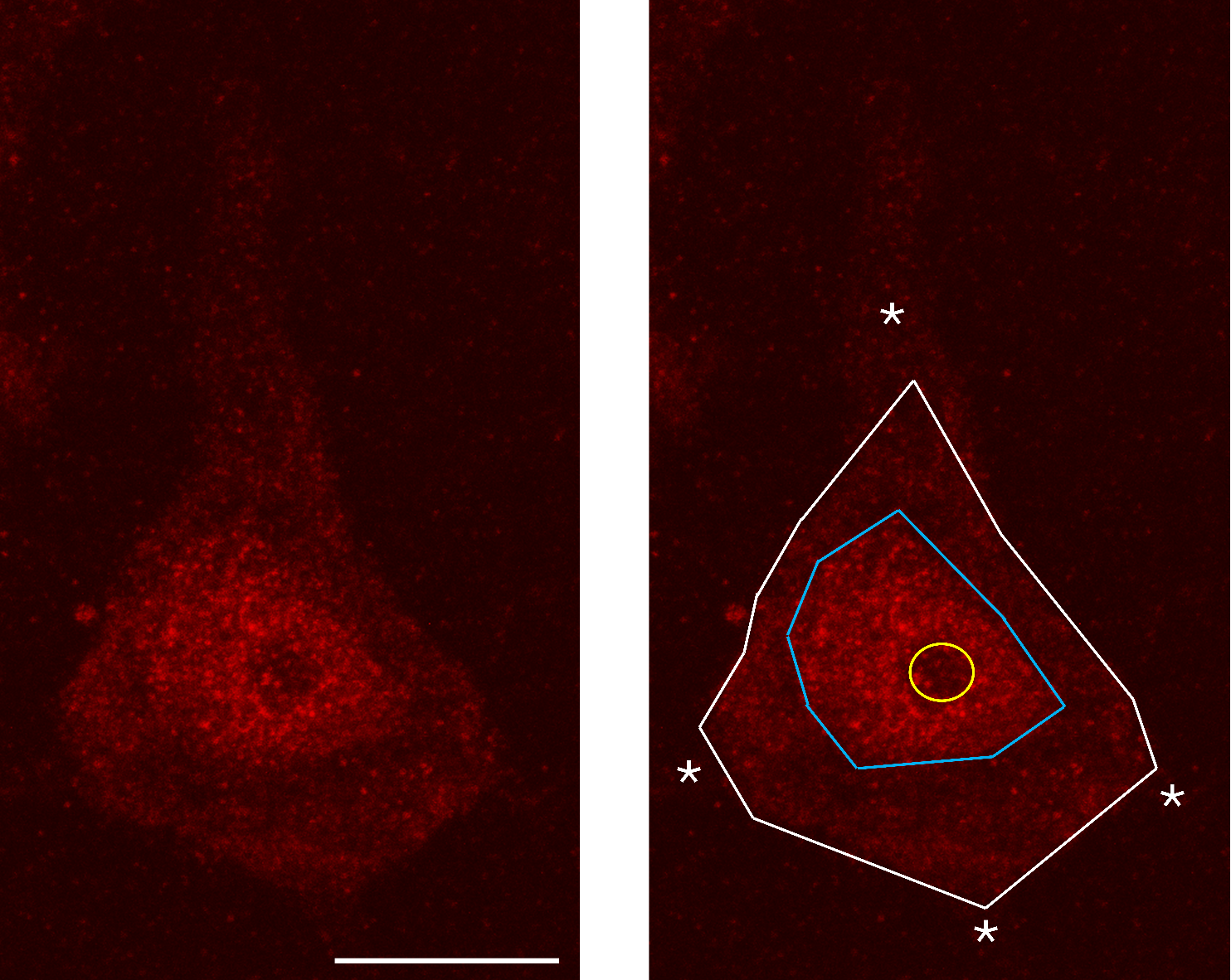


**Supplementary Figure 1**. NeuN-staining of a pyramidal neuron found in BA24. Scale bar: 10 µm. The right image shows the different degrees of NeuN-staining in different parts of the neuron. The soma outline is marked with the white contour. The intensely stained neuronal nucleus is marked with the blue contour. The nucleolus is marked with the yellow contour. The white asterisks (*) mark the faint NeuN-staining in the most proximal segments of the neuron’s cellular processes.


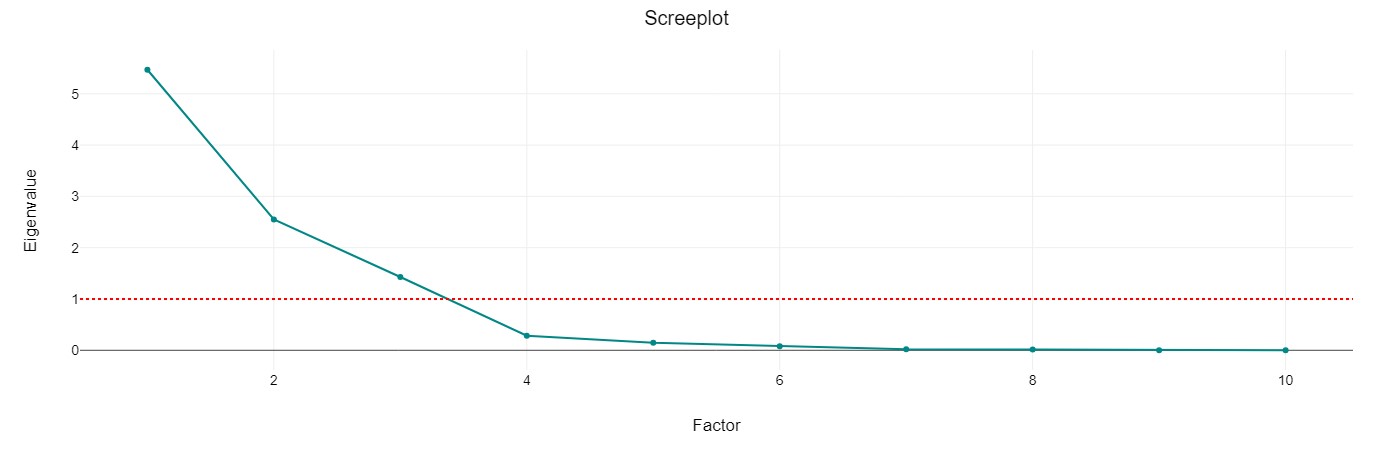


**Supplementary Figure 2.** Screeplot showing the relationship between the possible factors and the corresponding eigenvalues. The dashed red line denotes eigenvalue = 1.


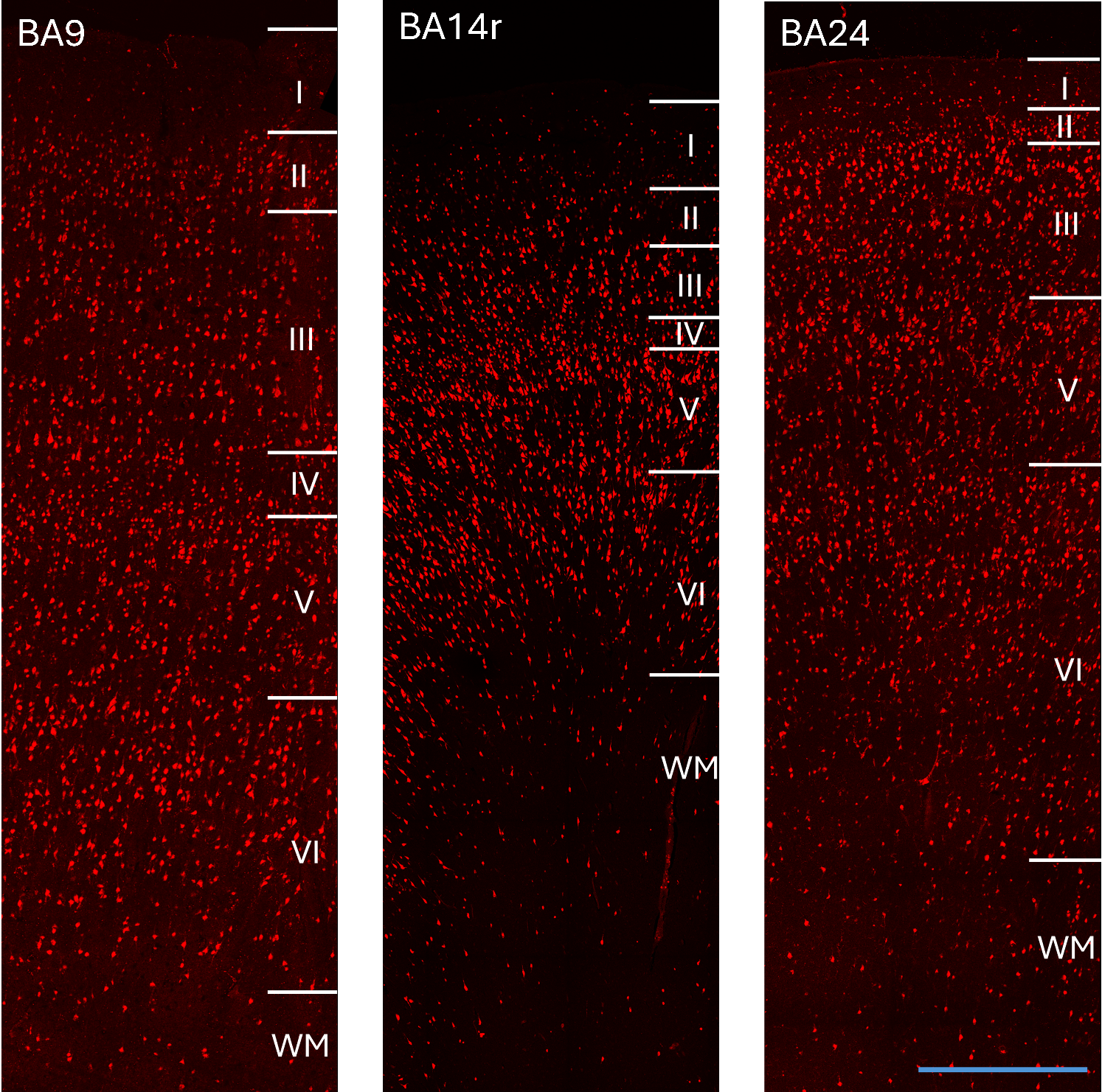


**Supplementary Figure 3.** Cortical columns showing the cytoarchitectonics of Brodmann areas 9, 14r, and 24, visualized using NeuN-staining. Note that NeuN-staining clearly shows the morphology of neuron cell bodies in the human cortex.

## Supplementary Tables

**Table S1.** Human brain tissue used in the research.

| **Subject** | **Sex** | **Age (years)** | **Postmortem interval (h)** | **Cause of death** | **Methodology** |
| --- | --- | --- | --- | --- | --- |
| CO382 | M | 40 | 6.5 | sudden cardiac death | immunofluorescence |
| CO383 | M | 51 | 11 | sudden cardiac death | immunofluorescence |
| CO384 | M | 46 | 6 | sudden cardiac death | immunofluorescence |
| CO386 | M | 37 | 6 | sudden cardiac death | immunofluorescence |
| CO387 | M | 44 | 6 | methadone/benzodiazepine overdose | immunofluorescence |

**Table S2.** Correlation matrix between different morphometric parameters. Values show Spearman’s correlation coefficient.

|  | *Perimeter* | *Area* | *Feret Max* | *Feret Min* | *Aspect Ratio* | *Compactness* | *Convexity* | *Form Factor* | *Roundness* | *Solidity* |
| --- | --- | --- | --- | --- | --- | --- | --- | --- | --- | --- |
| *Perimeter* | 1 | 0.99 | 0.98 | 0.91 | 0.29 | -0.41 | -0.25 | -0.50 | -0.41 | -0.37 |
| *Area* | 0.99 | 1 | 0.95 | 0.95 | 0.19 | -0.29 | -0.17 | -0.36 | -0.29 | -0.27 |
| *Feret Max* | 0.98 | 0.95 | 1 | 0.84 | 0.45 | -0.55 | -0.23 | -0.58 | -0.55 | -0.37 |
| *Feret Min* | 0.91 | 0.95 | 0.84 | 1 | -0.07 | -0.08 | -0.18 | -0.23 | -0.08 | -0.26 |
| *Aspect Ratio* | 0.29 | 0.19 | 0.45 | -0.07 | 1 | -0.93 | -0.13 | -0.68 | -0.93 | -0.25 |
| *Compactness* | -0.41 | -0.29 | -0.55 | -0.08 | -0.93 | 1 | 0.28 | 0.86 | 1 | 0.43 |
| *Convexity* | -0.25 | -0.17 | -0.23 | -0.18 | -0.13 | 0.28 | 1 | 0.52 | 0.28 | 0.71 |
| *Form Factor* | -0.50 | -0.36 | -0.58 | -0.23 | -0.68 | 0.86 | 0.52 | 1 | 0.86 | 0.69 |
| *Roundness* | -0.41 | -0.29 | -0.55 | -0.08 | -0.93 | 1 | 0.28 | 0.86 | 1 | 0.43 |
| *Solidity* | -0.37 | -0.27 | -0.37 | -0.26 | -0.25 | 0.43 | 0.71 | 0.69 | 0.43 | 1 |

**Table S3.** Rotated component matrix.

|  | Component | | |
| --- | --- | --- | --- |
|  | 1 | 2 | 3 |
| *Perimeter* | 0.25 | **-0.95** | -0.15 |
| *Area* | 0.09 | **-0.99** | -0.04 |
| *Feret Max* | 0.43 | **-0.89** | -0.1 |
| *Feret Min* | -0.16 | **-0.97** | -0.14 |
| *Aspect Ratio* | **0.95** | -0.07 | 0.02 |
| *Compactness* | **-0.96** | 0.18 | 0.17 |
| *Convexity* | -0.08 | 0.06 | **0.92** |
| *Form Factor* | -0.77* | 0.26 | 0.51* |
| *Roundness* | **-0.95** | 0.18 | 0.17 |
| *Solidity* | -0.28 | 0.18 | **0.87** |

**Table S4.** Percentage of excluded cells per cortical region and cortical layer.

|  | **Number of excluded cells** | **Total number of cells** | **Proportion of excluded cells (%)** |
| --- | --- | --- | --- |
| ***Cortical region*** |  |  |  |
| BA9 | 2854 | 36408 | 7.84 |
| BA14r | 1488 | 27328 | 5.44 |
| BA24 | 1684 | 24137 | 6.76 |
| ***Cortical layer*** |  |  |  |
| Layer I | 110 | 4537 | 2.42 |
| Layer II | 492 | 10934 | 4.50 |
| Layer III | 2173 | 30003 | 7.24 |
| Layer IV | 254 | 6774 | 3.75 |
| Layer V | 1349 | 18891 | 7.14 |
| Layer VI | 1648 | 16734 | 9.85 |
| ***Total*** | 6026 | 87873 | 6.86 |

**Table S5.** Descriptive statistics for morphometric parameters for cortical layers in the three analyzed cortical regions.

|  | BA | Layer | Mean ± SD | 95% CI for mean | CV | Median | IQR | Mode | Minimum | Maximum |
| --- | --- | --- | --- | --- | --- | --- | --- | --- | --- | --- |
| *Area* (µm²) | 9 | I | 76.95 ± 26.02 | 75.61 - 78.29 | 33.81% | 72.89 | 31.84 | 52.53 | 16.11 | 251.84 |
|  |  | II | 103.14 ± 49.68 | 101.64 - 104.64 | 48.17% | 91.22 | 60.20 | 44.52 | 12.19 | 362.82 |
|  |  | III | 184.58 ± 105.37 | 182.7 - 186.45 | 57.09% | 165.90 | 138.7 | 100.14 | 16.33 | 923.41 |
|  |  | IV | 114.15 ± 59.20 | 112.35 - 115.95 | 51.87% | 102.76 | 59.41 | 58.52 | 19.97 | 675.47 |
|  |  | V | 175.06 ± 105.58 | 172.69 - 177.43 | 60.31% | 145.05 | 132.48 | 56.97 | 21.05 | 809.85 |
|  |  | VI | 200.45 ± 92.16 | 198.43 - 202.48 | 45.97% | 194.10 | 133.14 | 51.76 | 14.36 | 635.92 |
|  | 14 | I | 75.23 ± 24.67 | 74.03 - 76.42 | 32.79% | 72.93 | 30.63 | 50.02 | 13.09 | 239.00 |
|  |  | II | 91.62 ± 40.21 | 90.35 - 92.89 | 43.89% | 83.91 | 47.31 | 52.44 | 20.88 | 361.56 |
|  |  | III | 161.38 ± 84.19 | 159.73 - 163.04 | 52.17% | 146.82 | 123.6 | 64.79 | 16.63 | 652.10 |
|  |  | IV | 103.12 ± 47.37 | 101.37 - 104.87 | 45.94% | 94.41 | 51.49 | 102.86 | 19.43 | 432.38 |
|  |  | V | 157.01 ± 89.72 | 154.7 - 159.33 | 57.14% | 131.42 | 118.29 | 65.66 | 17.85 | 561.90 |
|  |  | VI | 164.07 ± 74.76 | 161.8 - 166.35 | 45.57% | 156.57 | 107.16 | 76.29 | 16.91 | 650.58 |
|  | 24 | I | 76.77 ± 27.84 | 75.41 - 78.13 | 36.26% | 72.32 | 30.75 | 65.76 | 21.16 | 396.36 |
|  |  | II | 121.2 ± 61.87 | 119.06 - 123.34 | 51.05% | 107.63 | 85.14 | 160.22 | 15.35 | 502.04 |
|  |  | III | 176.85 ± 96.42 | 174.83 - 178.86 | 54.52% | 164.22 | 146.06 | 52.15 | 23.78 | 646.34 |
|  |  | IV | - | - | - | - | - | - | - | - |
|  |  | V | 205.18 ± 129.14 | 201.94 - 208.42 | 62.94% | 173.73 | 175.42 | 51.18 | 23 | 869.85 |
|  |  | VI | 207.77 ± 109.43 | 204.77 - 210.76 | 52.67% | 189.63 | 153.35 | 78.6 | 29.53 | 707.16 |
| *Aspect Ratio* | 9 | I | 1.43 ± 0.25 | 1.42 - 1.45 | 17.58% | 1.38 | 0.31 | 1.25 | 1.06 | 2.94 |
|  |  | II | 1.47 ± 0.27 | 1.46 - 1.48 | 18.56% | 1.41 | 0.33 | 1.22 | 1.04 | 3.33 |
|  |  | III | 1.52 ± 0.31 | 1.52 - 1.53 | 20.05% | 1.45 | 0.37 | 1.31 | 1.05 | 4.19 |
|  |  | IV | 1.43 ± 0.25 | 1.42 - 1.44 | 17.18% | 1.38 | 0.29 | 1.26 | 1.05 | 3.98 |
|  |  | V | 1.55 ± 0.31 | 1.54 - 1.56 | 20.22% | 1.48 | 0.38 | 1.38 | 1.05 | 3.9 |
|  |  | VI | 1.60 ± 0.35 | 1.59 - 1.61 | 21.83% | 1.53 | 0.42 | 1.49 | 1.06 | 4.03 |
|  | 14 | I | 1.45 ± 0.25 | 1.44 - 1.46 | 17.31% | 1.40 | 0.3 | 1.22 | 1.07 | 2.91 |
|  |  | II | 1.49 ± 0.26 | 1.48 - 1.49 | 17.50% | 1.44 | 0.34 | 1.40 | 1.04 | 2.97 |
|  |  | III | 1.57 ± 0.30 | 1.56 - 1.57 | 18.91% | 1.51 | 0.38 | 1.34 | 1.05 | 3.38 |
|  |  | IV | 1.45 ± 0.26 | 1.45 - 1.46 | 17.92% | 1.40 | 0.31 | 1.30 | 1.05 | 2.98 |
|  |  | V | 1.60 ± 0.35 | 1.59 - 1.61 | 22.17% | 1.52 | 0.43 | 1.38 | 1.07 | 3.99 |
|  |  | VI | 1.69 ± 0.41 | 1.67 - 1.70 | 24.20% | 1.60 | 0.50 | 1.35 | 1.05 | 4.52 |
|  | 24 | I | 1.42 ± 0.23 | 1.40 - 1.43 | 16.05% | 1.38 | 0.28 | 1.23 | 1.06 | 2.71 |
|  |  | II | 1.51 ± 0.30 | 1.50 - 1.52 | 19.85% | 1.45 | 0.36 | 1.26 | 1.07 | 3.72 |
|  |  | III | 1.56 ± 0.33 | 1.55 - 1.57 | 21.08% | 1.49 | 0.40 | 1.36 | 1.05 | 4.83 |
|  |  | IV | - | - | - | - | - | - | - | - |
|  |  | V | 1.69 ± 0.57 | 1.67 - 1.70 | 33.55% | 1.55 | 0.48 | 1.34 | 1.07 | 7.60 |
|  |  | VI | 1.62 ± 0.41 | 1.61 - 1.64 | 25.06% | 1.53 | 0.45 | 1.40 | 1.07 | 5.85 |
| *Form Factor* | 9 | I | 0.88 ± 0.07 | 0.87 - 0.88 | 7.54% | 0.89 | 0.08 | 0.91 | 0.57 | 0.98 |
|  |  | II | 0.85 ± 0.08 | 0.85 - 0.85 | 9.30% | 0.87 | 0.10 | 0.88 | 0.48 | 0.98 |
|  |  | III | 0.80 ± 0.10 | 0.80 - 0.80 | 12.30% | 0.82 | 0.14 | 0.85 | 0.34 | 0.98 |
|  |  | IV | 0.86 ± 0.08 | 0.86 - 0.86 | 8.96% | 0.87 | 0.10 | 0.93 | 0.45 | 0.98 |
|  |  | V | 0.82 ± 0.09 | 0.81 - 0.82 | 11.39% | 0.83 | 0.13 | 0.85 | 0.31 | 0.98 |
|  |  | VI | 0.81 ± 0.10 | 0.80 - 0.81 | 11.79% | 0.82 | 0.13 | 0.89 | 0.38 | 0.98 |
|  | 14 | I | 0.87 ± 0.07 | 0.87 - 0.87 | 7.75% | 0.88 | 0.08 | 0.89 | 0.43 | 0.98 |
|  |  | II | 0.86 ± 0.07 | 0.85 - 0.86 | 8.24% | 0.87 | 0.10 | 0.88 | 0.55 | 0.98 |
|  |  | III | 0.81 ± 0.09 | 0.81 - 0.81 | 10.92% | 0.82 | 0.12 | 0.80 | 0.39 | 0.98 |
|  |  | IV | 0.86 ± 0.07 | 0.86 - 0.86 | 8.54% | 0.88 | 0.10 | 0.90 | 0.50 | 0.98 |
|  |  | V | 0.81 ± 0.09 | 0.81 - 0.81 | 11.41% | 0.82 | 0.13 | 0.85 | 0.37 | 0.98 |
|  |  | VI | 0.80 ± 0.10 | 0.80 - 0.81 | 12.18% | 0.81 | 0.14 | 0.80 | 0.32 | 0.98 |
|  | 24 | I | 0.87 ± 0.06 | 0.87 - 0.88 | 7.42% | 0.89 | 0.08 | 0.91 | 0.59 | 0.98 |
|  |  | II | 0.85 ± 0.08 | 0.85 - 0.86 | 9.43% | 0.87 | 0.10 | 0.91 | 0.38 | 0.98 |
|  |  | III | 0.82 ± 0.09 | 0.82 - 0.83 | 10.72% | 0.84 | 0.12 | 0.85 | 0.34 | 0.98 |
|  |  | IV | - | - | - | - | - | - | - | - |
|  |  | V | 0.80 ± 0.11 | 0.80 - 0.81 | 13.45% | 0.82 | 0.13 | 0.82 | 0.23 | 0.98 |
|  |  | VI | 0.81 ± 0.1 | 0.81 - 0.81 | 11.85% | 0.82 | 0.13 | 0.88 | 0.30 | 0.98 |

**Table S6.** Morphometric parameters for each morphological cell type.

|  | Cell type | Mean ± SD | 95% CI for mean | Median | IQR | Mode | Minimum | Maximum |
| --- | --- | --- | --- | --- | --- | --- | --- | --- |
| *Area* (µm²) | Granule | 74.26 ± 29.67 | 72.86 - 75.66 | 68.52 | 32.38 | 55.33 | 13.09 | 321.09 |
|  | Small pyramidal | 141.15 ± 47.28 | 135.1 - 147.21 | 133.61 | 61.07 | 155.04 | 48.64 | 339.41 |
|  | Medium pyramidal | 291.43 ± 86.8 | 283.6 - 299.25 | 274.67 | 104.42 | 196.94 | 95.6 | 707.1 |
|  | Large pyramidal | 460.36 ± 125.83 | 440.06 - 480.66 | 446.88 | 126.07 | 92.93 | 92.93 | 959.66 |
|  | Small fusiform | 108.81 ± 48.46 | 96.29 - 121.33 | 99.92 | 47.67 | 41.13 | 41.13 | 313.48 |
|  | Large fusiform | 306.26 ± 110.88 | 288.07 - 324.46 | 286.51 | 136.71 | 141.95 | 141.95 | 861.5 |
|  | Polymorphic | 141.23 ± 103.07 | 112.21 - 170.24 | 110.29 | 84.95 | 46.65 | 46.65 | 659.35 |
| *Aspect Ratio* | Granule | 1.32 ± 0.16 | 1.31 - 1.33 | 1.29 | 0.21 | 1.23 | 1.05 | 1.96 |
|  | Small pyramidal | 1.56 ± 0.26 | 1.53 - 1.6 | 1.52 | 0.33 | 1.45 | 1.13 | 2.61 |
|  | Medium pyramidal | 1.63 ± 0.26 | 1.6 - 1.65 | 1.59 | 0.32 | 1.48 | 1.1 | 2.78 |
|  | Large pyramidal | 1.67 ± 0.34 | 1.62 - 1.73 | 1.61 | 0.35 | 1.63 | 1.16 | 2.99 |
|  | Small fusiform | 2.44 ± 0.43 | 2.33 - 2.55 | 2.38 | 0.54 | 2.33 | 1.76 | 3.87 |
|  | Large fusiform | 2.4 ± 0.48 | 2.32 - 2.48 | 2.35 | 0.69 | 2.11 | 1.33 | 3.74 |
|  | Polymorphic | 1.8 ± 0.46 | 1.67 - 1.93 | 1.73 | 0.61 | 1.37 | 1.21 | 3.4 |
| *Form Factor* | Granule | 0.92 ± 0.04 | 0.92 - 0.92 | 0.93 | 0.04 | 0.94 | 0.75 | 0.98 |
|  | Small pyramidal | 0.78 ± 0.07 | 0.77 - 0.79 | 0.79 | 0.09 | 0.79 | 0.53 | 0.93 |
|  | Medium pyramidal | 0.75 ± 0.07 | 0.74 - 0.75 | 0.75 | 0.10 | 0.79 | 0.45 | 0.94 |
|  | Large pyramidal | 0.73 ± 0.07 | 0.72 - 0.74 | 0.74 | 0.09 | 0.75 | 0.54 | 0.89 |
|  | Small fusiform | 0.70 ± 0.07 | 0.68 - 0.70 | 0.69 | 0.09 | 0.73 | 0.55 | 0.86 |
|  | Large fusiform | 0.69 ± 0.08 | 0.68 - 0.70 | 0.70 | 0.13 | 0.71 | 0.55 | 0.91 |
|  | Polymorphic | 0.69 ± 0.08 | 0.66 - 0.71 | 0.70 | 0.11 | 0.63 | 0.45 | 0.86 |
| *Solidity* | Granule | 0.99 ± 0.01 | 0.99 - 0.99 | 1.00 | 0.01 | 1.00 | 0.86 | 1.00 |
|  | Small pyramidal | 0.97 ± 0.02 | 0.97 - 0.97 | 0.98 | 0.03 | 0.99 | 0.89 | 1.00 |
|  | Medium pyramidal | 0.96 ± 0.03 | 0.96 - 0.96 | 0.97 | 0.03 | 0.98 | 0.85 | 1.00 |
|  | Large pyramidal | 0.96 ± 0.03 | 0.95 - 0.96 | 0.97 | 0.04 | 0.97 | 0.87 | 1.00 |
|  | Small fusiform | 0.97 ± 0.03 | 0.96 - 0.98 | 0.98 | 0.03 | 0.99 | 0.90 | 1.00 |
|  | Large fusiform | 0.97 ± 0.03 | 0.97 - 0.98 | 0.98 | 0.03 | 0.99 | 0.87 | 1.00 |
|  | Polymorphic | 0.91 ± 0.04 | 0.90 - 0.92 | 0.91 | 0.06 | 0.94 | 0.80 | 0.98 |

# Video material and 3D reconstructions

All video materials and accompanying reconstruction files are 3D reconstructions from histological sections taken from BA24. The sections were processed using anti-NeuN immunofluorescent staining (red in all videos) and imaged using a confocal microscope (100x objective). The entire thickness of the section was imaged using a Z-stack, and the distance between individual Z-slices was 1 µm.

**01 Pyramidal cell** – 3D reconstruction (transparent light blue) of a pyramidal cell.

**02 Granule cell** – 3D reconstruction (transparent light blue) of a granule cell.

**03 Fusiform cell** – 3D reconstruction (transparent light blue) of a fusiform cell.
